# Assessing the Effect of Spiritual Intelligence Training on Spiritual Care Competency in Critical Care Nurses

**DOI:** 10.25122/jml-2018-0056

**Published:** 2018

**Authors:** Somayeh Riahi, Fateme Goudarzi, Shirin Hasanvand, Hasan Abdollahzadeh, Farzad Ebrahimzadeh, Zahra Dadvari

**Affiliations:** 1. Student Research Committee, School of Nursing and Midwifery, Lorestan University of Medical Sciences, Khorramabad, Iran; 2. Social Determinants of Health Research Center, Lorestan University of Medical Sciences, Khorramabad, Iran; 3. Department of Psychology, Payame Noor University, Tehran, Iran; 4. Shahid Rahimi Hospital, Khorramabad, Iran

**Keywords:** Spiritual Care, Spiritual Care Competency, Spiritual Intelligence, Training, Education, Nurse

## Abstract

**Aim & Objective**: Due to the importance of spiritual care as a part of holistic care, this study aimed to investigate the effect of spiritual intelligence training on the nurses’ competence in spiritual care in critical care units.

**Methods:** The study was performed on 82 nurses (40 in the experimental group and 42 in the control group). Participants were selected from critical care units of teaching hospitals affiliated to Lorestan University of Medical Sciences^1^. The experimental group took part in eight sessions of spiritual intelligence training, held in the form of workshops. In the control group, no intervention was made. The scale for assessing nurses’ competencies in spiritual care was completed before, immediately and one month after the sessions in two groups. Data analysis was performed using SPSS software version 15.

**Results:** The results showed that spiritual intelligence training had a positive effect on nurses’ competence in spiritual care. Also, 89% of the nurses who participated in the study had not been given any prior education regarding spiritual care. Nurses considered barriers to spiritual care including inadequate staff, cultural differences, high workload and lack of education on this subject.

**Conclusions:** The present results showed that the training of spiritual intelligence could develop the nurses’ competence in spiritual care. The development of spiritual care provided by nurses can result in various outcomes such as increased satisfaction with care in patients, reduced anxiety and symptoms of depression during hospitalization, reduced length of hospitalization and, in general, improved quality of life.

## Introduction

A holistic approach encourages nurses to address all the physical, mental, emotional, spiritual and social needs of the patient by providing a complete model of care [1]. Meanwhile, spiritual needs are the deepest needs of the individual, and if nurses spend more time dealing with the spiritual issues of the patients, they will help resolve the psychological and physical problems of the patients [2]. On the other hand, many patients demand the attention of doctors, nurses and other health workers to spiritual issues and wish to discuss spiritual-related topics [3]. Accordingly, spiritual care is also provided to meet the spiritual needs of the patient [4], help people search for meaning and goals, and to show the unconditional acceptance of the patient-nurse and promote hope and relaxation [5].

Cavendish et al. (2003) believed that spiritual care improves health and has positive effects on people’s stress, self-esteem, and perfectionism as well as interpersonal relationships [6]. On the other hand, review of literature shows that the involvement of spiritual care in the treatment has some results, such as symptoms’ recovery and reduction of disease relapse [7], increased satisfaction with care [8], decreased symptoms of depression [9], decreased need for invasive interventions at the end stage of life, decreasing the costs and hospitalization length [10], and generally improving quality of life [11].

In the meantime, patients in critical care units require more mental and spiritual considerations because on the one hand, these patients are faced with some critical situations such as the sense of approaching death, suffering, isolation, fear, vulnerability and complete dependence and on the other hand, today’s modern approaches, which are disease-focused, have replaced the patient-focused approach in critical care units [12].

It is worth noting that nurses need special skills such as self-awareness, communication, trust, hope and to be a catalyst for spiritual growth in order to provide spiritual care and achieve optimal results in patients’ health [13]. According to Pellebon and Anderson (1999), spirituality has a significant impact on attitudes, behaviors and the individual’s decisions [14]. In addition, the spiritual self-awareness of nurses improves the provision of spiritual care [15]. Therefore, spiritual intelligence as a deep self-awareness can lead to improvements in the provision of this kind of care [16].

Spiritual intelligence is a framework for identifying and organizing the skills and performance compatibilities needed to use spirituality and can develop an individual’s ability to solve problems and achieve goals [17]. Faith, humility, appreciation, the ability to integrate, to control one’s feelings, ethics and ethical behavior, forgiveness and love are characteristics of spiritual intelligence [18]. Wigglesworth (2002) believes that spiritual intelligence is the ability to deal with compassion and wisdom with inner and outer calmness “under any circumstances.” Compassion and wisdom both represent love, and the term “under any circumstances” suggests that we can have calmness and compassionate behaviors in any tense environment. He also argues that the advancement of spiritual intelligence is beneficial not only for individuals but also for their families, communities and their work [19]. Peace of mind, mutual understanding, agreement among colleagues and job satisfaction are other benefits of using spiritual intelligence in the workplace [20]. Findings of Kaur et al.’s (2013) study have also shown the effect of spiritual intelligence on emotional intelligence and nursing care behaviors by presenting a model of caring behaviors among nurses [21]. Regarding the importance of spiritual care and the relationship between spiritual intelligence and nurses’ caring behaviors, this study examined the effect of spiritual intelligence training on the nurses’ competence in spiritual care (NCSC) in critical care units.

## Background

While many studies talk about the positive attitude of nurses towards spirituality and spiritual care [22, 23], evidence of the nurses’ competence in providing spiritual care presents different findings. The study by Attard et al. (2014) reported the desirability of nursing students in providing spiritual care [24]; however, the results of the study by Adib Hajbagheri and Zehtabchi (2017) indicated that nurses did not have a good professional competence in providing spiritual care due to the lack of education in this regard [25]. Also, in a study conducted by Balboni et al. (2014), it was clarified that although most nurses and doctors (76%) attempted to provide spiritual care, only 39% of them managed to provide it. This research reported the lack of time and inadequate education as causes of the weaknesses in providing spiritual care [26].

Lundberg (2010) acknowledged that in order to provide holistic care, nursing education programs should increase nurses’ understanding and awareness of spiritual issues in order to meet the spiritual needs of patients [27]. However, a review of the literature suggests that nurses do not receive adequate education regarding spiritual care [28–30]. Studies on competence in spiritual care are mostly descriptive, and the evidence which is aimed at improving the nurses’ competence is limited. In line with factors affecting competence in spiritual care, Ross et al. (2016) stated that the personal spirituality of nursing students is effective in understanding their competence in spiritual care [15].

As presented in the introduction section, spiritual intelligence can be considered a factor in spiritual care because it can improve self-awareness in a person. Spiritual intelligence can be strengthened independently and augmented through search, inquiry, and practice [31]. Although no studies were done on the effect of spiritual intelligence training on competence in spiritual care, in this regard, descriptive studies show that there is a positive relationship between spiritual intelligence and health [32], happiness [33] and resilience of nurses [34]. Therefore, considering the importance of spiritual care in the quality of care, the present study titled “Assessing the Effect of Spiritual Intelligence Training on Spiritual Care Competency in Critical Care Nurses” was conducted.

## Method

Participants: In the present study, the research population included nurses working in critical care units of teaching hospitals affiliated to LUMS. The sample size was calculated based on the formula 
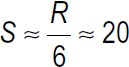
 with α = 0.05, and β = 0.1 and d = 15, in about 37 people. Considering the probability of 10% loss of samples, 45 people were considered as the sample size of each group.

**Figure:**
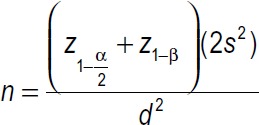


At first, three teaching hospitals affiliated to LUMS in Khorramabad city were selected as population clusters. Then, by using a randomized systematic method, Shahid Madani and Shahid Rahimi hospitals were selected from among the clusters based on the map of Khorramabad. Initially, by convenience sampling method, 45 nurses who were admitted to the critical care units and volunteered to participate in the study were selected. After explaining the research objectives and securing confidentiality of information, they were entered into the experimental group. Then, 45 nurses were elected for the control group by matching them with the experimental group by age, gender, educational degree and employment status. Informed consent was taken from all participants. The criteria for entering nurses into the study included employment in the critical care units with a minimum of a year’s work experience, with at least Bachelor’s degree in nursing and no history of participation in spiritual intelligence training courses or similar research. Exclusion criteria also included a reluctance to participate and continued collaboration in conducting research and not attending training sessions for more than one session. In this study, five nurses of the experimental group were excluded due to their absence in more than one session, and in the control group, three questionnaires were excluded due to being incomplete.

### Design

The study aimed to assess the effect of spiritual intelligence training on the nurses’ competence in spiritual care (NCSC) in critical care units. It has been designated as a semi-experimental study with two-groups with a pretest-posttest design. The present study was conducted in two teaching hospitals of LUMS, Shahid Madani and Shahid Rahimi. The spiritual intelligence training protocol was compiled using the components of Emmons (2000), a book by Dr. Hassan Abdullah Zadeh titled as “Spiritual Intelligence” (in Persian) [35] and studies on spiritual intelligence training [36–38]. The content of the educational package was confirmed by a specialist in this field (Dr. Hassan Abdullah Zadeh, who has a history of writing a book and presenting an article as well as creating tools for measuring spiritual intelligence). The training sessions were conducted by the researcher in a workshop for eight weeks (eight sessions of 90 minutes each). The concepts presented to participants in the sessions are given in Table 1. During each session, the concepts were first described and then all the participants were involved in practical exercises in the training session as well as at home. Also, at the end of each session, the training programs for relaxation and meditation were presented to the participants on a hard-disk for practicing relaxation at home. For easy access to participants, training sessions were held, and questionnaires were completed in the hospitals.

**Table 1:** Number, titles and content of the eight sessions of the Spiritual Intelligence Training Workshop

Sessions	Session titles	Goals
First session	Familiarity of researcher and learners; explanations on spiritual care and spiritual intelligence	Explaining the goals of the research; setting up the sessions; familiarity with the concepts of spiritual care and spiritual intelligence
Second session	Spiritual self-awareness	Understanding self-awareness and raising self-awareness among participants
Third session	The meaning of life	Understanding the meaning of daily activities, even in the event of suffering
Fourth Session	Problem-solving with a spiritual approach	Awareness of the usefulness of the spiritual approach in problem-solving
Fifth session	The skill of forgiveness in restoring interpersonal relationships	Applying forgiveness skills to improve your relationships
Sixth session	To learn some religious words or expressions (Rosary)	Understanding a greater calmness rosary and prayer
Seventh session	Evaluation and focus on commitment	Understanding your values and commitment to a healthy life
Eighth session	Insight and mission	Increasing intuition and insight into the missions they are considering for themselves in this world

Data collection tools were the Scale for Assessment of the Nurse’s professional Competence in Spiritual Care (SANCSC) and the King’s Spiritual Intelligence Scale. To prevent contamination, the control group completed the questionnaires first and after those training sessions were held for the experimental group. The nurses’ competence was assessed in the experimental group before beginning the training sessions by using the two above-mentioned tools, the King’s Spiritual Intelligence Scale and SANCSC. Once again, immediately after the sessions, as well as a month later, two questionnaires were completed by the experimental group. In the control group, the time intervals would be as follows: The first time was after taking informed consent (as a baseline), the second one would be eight weeks after the first one, and the third one would be one month after the second one.

### Instrument

Data collection was done by using two questionnaires including the Scale for Assessment of the Nurse’s Professional Competence in Spiritual Care (SANCSC) and the King’s Spiritual Intelligence Scale.

SANCSC has been made by Adib Hajbagheri and Zehtabchi (2014) based on Iranian culture, and it consists of three parts. The first part consists of demographic and occupational information including nine items pertaining to age (year), gender (male and female), marital status (single, married), work experience (years), educational level (undergraduate and postgraduate), employment status (permanent, contractual, contract and internship), the workplace (CCU^2^ and ICU^3^), the nurse’s position (nurse and head nurse), and previous training on spiritual care (yes and no). The second part consists of a questionnaire for assessing the nurse’s professional competence in spiritual care, consisting of 32 items in five areas including assessment and implementation of spiritual care (17 items), human values (six items), knowledge (four items), attitude (three items) and self-recognition (two items). The items in this questionnaire are answered in the four-point Likert scale (always = 5, often = 4, sometimes = 3, rarely = 2, never = 1) and self-report. The total score of this questionnaire is in the range of 32 to 160, and scores 118 and higher indicate good competence of spiritual care, scores 117-74 represent average competence and scores less than 73 show weak competence. The third part of the questionnaire includes an open question “in your opinion, what are the barriers to providing spiritual care for patients?”

The content validity of the questionnaire was evaluated by 20 experts and computed by the Content Validity Index (CVI) and Content Validity Ratio (CVR). The CVI for items in the domain of relevance was in the range of 0.87 to 1, the simplicity ranged from 0.8 to 1, the clear range was 0.8 to 1, and CVR ranged from 0.6 to 1. The entire CVI for the questionnaire in three areas of relevance, simplicity, and clarity was 0.963, 0.936, and 0.857, respectively. Also, the reliability of the questionnaire was investigated by internal consistency and Cronbach’s alpha calculation, which for the whole method was equal to 0.941. For the assessment and implementation of the spiritual care it was 0.931, the domain of human values was 0.836, the knowledge domain was 0.837, while the scope of the attitude was 0.827 and for the self-recognition domain was 0.758 [25, 39].

The King’s Spiritual Intelligence Scale by Raghib et al. (2010) in Iran is validated, and its reliability estimated using Cronbach’s alpha coefficient was 0.88. Face and content validity was confirmed by psychologists. In order to estimate the convergent validity, Ghobari Banab’s Spiritual Experiences Scale (SES) (2005) was used simultaneously, and the correlation coefficients of these two questionnaires were 0.66. Also, for calculating the construct validity, the exploratory factor analysis and first-order confirmation factor analysis were calculated. The spiritual intelligence scale has 24 items in the four subscales of critical existential thinking (five items), personal meaning production (seven items), transcendental awareness (five items), and the conscious state expansion (seven items). This scale is ranked in a four-point Likert scale (0 = no comment, 1 = not true, 2 = somewhat correct, 3 = very correct, 4 = perfectly correct). Only the sixth item will be scored reversely [40].

### Ethical Consideration

At first, the authorization was received from the Ethics Committee of LUMS. Oral permission was obtained from the participants and they were assured that the results of the study were confidential. They were also able to withdraw from the study whenever they stopped wanting to participate. In coordination with the continuing education officer, a retraining grade was obtained for the nurses of the test group. After the end of the training, the contents of the training were provided to the control group on a flash memory. Using the instruments was permitted by their developers.

### Data Analysis

The collected data were analyzed using SPSS software version 15. For data analysis, descriptive statistics (mean and standard deviation) and analytical statistical methods were used. Univariate tests consisted of chi-square, Fisher’s exact test, Independent t-test and One-way ANOVA, which was used to compare two groups in terms of demographic and underlying characteristics. Those variables that had a p-value of less than 0.25 in the univariate study were included in the multivariate analysis as a confounder [41, 42]. The ANCOVA model was used to modify the confounders (with a significant level of 0.05). Repeated Measure was used to compare the two groups in terms of mean scores before, immediately after and one month after the study. Content analysis was used to analyze open-answer questions.

### Findings

This study was performed on 82 nurses, 42 of whom were in the control group and 40 in the experimental group. There was no significant difference between the two groups regarding age, gender, work experience, education degree, employment status, the name of the working unit and previous education on spiritual care (p> 0.05). Also, in evaluating the mean scores of spiritual care competence and its areas before the study, it was found that there was no significant difference between the two groups (p> 0.05) (Table 2).

**Table 2:** Frequency distribution of the studied units in two groups in terms of occupational and demographic variables

Variable name*	Category	Experimental group n = 40	Control group n = 42	p-value**
Age	Under 40 years	33 (82.5%)	35 (83.3%)	>0.999
	Over 40 years	7 (17.5%)	7 (16.7%)	
Gender	Female	37 (92.5%)	39 (92.9%)	>0.999
	Male	3 (7.5%)	3 (7.1%)	
Work Experience	Less than 5 years	8 (20.0%)	10 (23.8%)	0.917
	Between 5-14 years	25 (62.5%)	25 (59.5%)	
	More than 14 years	7 (17.5%)	7 (16.7%)
Education degree	Bachelor	35 (87.5%)	36 (85.7%)	>0.999
	Masters	5 (12.5%)	6 (14.3%)	
Employment Status	Official/Petition	33 (82.5%)	36 (85.7%)	0.768
	Contractor/design	7 (17.5%)	6 (14.3%)	
Workplace	CCU	17 (42.5%)	23 (54.8%)	0.280
	ICU	23 (57.5%)	19 (45.2%)	
Previous training for spiritual care	Yes	6 (15.0%)	3 (7.1%)	0.307
	No	34 (85.0%)	39 (92.9%)	
Evaluation and implementation of spiritual care (before study)	—	54.65±4.05	53.30±4.06	0.139
Human values (before study)	—	19.70±1.65	19.52±1.77	0.642
Knowledge (before study)	—	13.82±1.59	13.35±1.47	0.173
Attitudes (before study)	—	12.22±1.49	11.78±1.00	0.124
Self-recognition (before study)	—	6.50±0.87	6.69±0.86	0.326
Competence in spiritual care (before study)	—	106.90±6.40	104.66±5.69	0.100

* For qualitative variables, the frequency (%) and for quantitative variables were used as mean ± standard deviation.

** For qualitative variables, Fisher’s exact test and Chi-square were used and T-test was used for quantitative variables.

Table 3 shows the mean scores of nurses’ spiritual intelligence in both the experimental and control groups over time. As shown in the table, the covariance analysis (by adjusting the effect of the values of the base of spiritual intelligence) showed that the main effect of intervention on the mean scores of spiritual intelligence was significant (p<0.05), so that after the study, the mean scores of spiritual intelligence were 78.50 in the experimental group from study. The repeated measures analysis showed that the main effect of time on the mean scores of spiritual intelligence was not significant (p>0.05). As a result, these values have not changed considerably over time. The repeated measures analysis showed that the interaction between time and group on the mean scores of spiritual intelligence was not significant (p>0.05). That is, the difference in spiritual intelligence score between the two groups over time did not change significantly (Table 3).

**Table 3:** Mean scores of King’s spiritual intelligence scale in two groups (experimental and control) over the time

Group	Before the study	Immediately after study	A month after study	p-value^1^	p-value^2^	p-value^3^
	Mean ±standard deviation	Mean ±standard deviation	Mean ±standard deviation			
Experimental group	53.37±5.17	78.50±4.99	79.25±5.60	<0.001	0.297	0.723
Control group	51.64±5.50	52.64±6.41	52.76±6.43			

1 Covariance analysis test to investigate the effect of intervention

2 Repeated measurement tests to check the effect of time

3 Repeated measurement tests to examine the interaction of time-group

Table 4 shows the mean scores of spiritual care competence and its areas in the control and experimental groups. The covariance analysis test (by adjusting the effect of the values of the base of spiritual care competence, spiritual intelligence, previous education on spiritual care and work unit) showed that the main effect of an intervention on the mean scores of spiritual care competence and its areas was significant (p<0/05). Therefore, after the study, the mean scores of spiritual care competence and its areas in the experimental group were higher than the control group. The repeated measures analysis showed that the main effect of time on the mean scores of spiritual care competence and its areas was not significant (p> 0.05). As a result, these values have not changed considerably over time. Only in human values there was a significant difference between times from the mean scores’ perspective, and these values increased one month later. Also, repeated measures analysis showed that the interaction between time and group on the mean scores of spiritual care competence was not significant (p>0.05). This means that the difference in the scores of spiritual care competence and its areas between the two experimental groups over time did not change significantly (Table 4).

**Table 4:** The mean scores of spiritual care competence and its areas in the two groups over time

The areas of spiritual care competence	Group	Before the study	Immediately after reading	A month after study	p-value^1^	p-value^2^	p-value^3^
		Mean ± standard deviation	Mean ± standard deviation	Mean ± standard deviation			
Evaluation and implementation of spiritual care	Experimental	54.65±4.05	69.15±3.81	69.27±3.10	<0.001	0.339	0.214
	Control	53.30±4.06	52.21±5.51	53.45±4.41			
Human values	Experimental	19.70±1.65	23.82±1.63	24.20±1.95	<0.001	0.037	0.309
	Control	19.52±1.77	19.61±1.79	19.50±1.58			
Awareness	Experimental	13.82±1.59	16.95±0.90	17.25±1.25	<0.001	0.293	0.318
	Control	13.35±1.47	13.11±1.41	13.14±1.52			
Attitudes	Experimental	12.22±1.49	14.10±0.54	13.97±0.97	0.005	0.371	0.653
	Control	11.78±1.00	11.78±1.11	11.71±1.08			
Self-recognition	Experimental	6.50±0.87	8.30±0.72	8.30±0.82	0.014	0.987	0.558
	Control	6.69±0.86	6.64±0.79	6.64±0.82			
Competence in spiritual care	Experimental	106.90±6.40	132.32±4.96	133.00±4.54	<0.001	0.369	0.374
	Control	104.66±5.69	103.38±7.54	104.45±6.63			

1. Covariance analysis test to investigate the effect of intervention

2. Repeated measurement tests to check the effect of time

3. Repeated measurement tests to examine the interaction of time-group

Also, Fisher’s exact test showed that there was no significant difference between the two groups in terms of the barriers to providing spiritual care competence. Regarding nurses, these barriers include insufficient time during a shift (54.9%), inadequate staff (46.3%), cultural difference (32.9%), high workload (31.7%), lack of nurse training (25.6%), low nursing motivation (22%), patient’s barriers (18.3%), inadequate nursing skills (11%), lack of facilities in the unit (9.8%), lack of familiarity with their spiritual needs (8.5%), lack of adaptation plan (4.9%) and language barrier (4.9%).

## Discussion

The findings of the study indicated that the mean scores of nurses’ competence in spiritual care (NCSC) in both groups was moderate before the study. On the other hand, in the experimental group, after training spiritual intelligence, the competence of spiritual care increased significantly, indicating that spiritual intelligence training was effective on the competence of spiritual care. The results of the study by Adib Hajbagheri and Zehtabchi also showed that the nurses’ competence in spiritual care (NCSC) was moderate [25]. However, Attard et al. (2014), offered students the desire to provide spiritual care [24]. This difference in the competence of spiritual care in students and nurses can be attributed to the importance given by universities’ nursing courses to the category of spirituality and spiritual care in recent years.

On the other hand, nurses received about 80 percent of score of attitude to spirituality and spiritual care indicating that they have a good attitude toward spiritual care. Investigating evidence also indicated a positive attitude of nurses toward spiritual care [22, 23]. However, 89 percent of the participating nurses said they had not received training on spiritual care. In a study by Wu et al. (2016), it was also found that 87.5% of participants did not receive proper education and expressed the need for further education [22]. In this regard, many studies have found that nurses are not adequately trained in this field, which is one of the important reasons for neglecting spiritual care [12, 25, 28].

Considering the lack of adequate training of nurses regarding spirituality and spiritual care, the results of this study indicate that the inclusion of spiritual intelligence in nursing training can help nurses to pay more attention to the spiritual needs of patients.

Although an empirical study to improve the nurses’ competence in spiritual care (NCSC) was not found, studies related to spiritual intelligence training and nursing care, such as Kaur et al. (2013), show that spiritual intelligence is a key element of nursing care behaviors [21]. As already mentioned, the competence of spiritual care as part of holistic care has a great positive effect on improving the health and quality of life of the patients [11]. Other descriptive studies also show a positive relationship between spiritual intelligence and health [32] and nurses’ happiness [33]. The study of Charkhabi et al. (2014) also showed that with the provision of spiritual intelligence training, the mental health of students increased [37].

Participating nurses considered barriers to providing spiritual care including inadequate shifts, inadequate staff, cultural differences, high workload, lack of nursing education, lack of motivation, inadequate nursing skills, lack of facilities, lack of understanding of spiritual needs, non-compliance with the adaptation plan and the language barrier. Nurses considered factors such as lack of managerial support, lack of encouragement from authorities, lack of periodic breaks and timely payments to be a cause for their lack of motivation. A study by McBrien et al. (2010) suggests factors such as lack of nursing personnel, lack of awareness of spiritual needs and time constraints as barriers to spiritual care provision [43]. Wong et al. (2010) also highlighted cultural differences, high workload, lack of managerial support, lack of knowledge and skills in providing spiritual care as obstacles to providing spiritual care [44].

Studies show that nurses-patients ratio of job burnout and job dissatisfaction in nurses [45] and overall quality of care are effective [46]. In the present study, it was also found that the disparity in the number of nurses and patients affected the spiritual care aspect. In this study, the cultural difference has had little effect, and perhaps because in recent years, in order to eliminate cultural differences, nurses in each region are trained and employed there.

## Conclusions

Given that many of the barriers to providing spiritual care can be addressed, identifying and removing these factors can be helpful in improving the provision of this type of care and, subsequently, improving the patients’ health. As mentioned, one of these obstacles is the lack of proper training in this subject that nursing managers should pay more attention to. The limitations of this study are a small number of participants and researching in a restricted geographic area. Therefore, the necessity of further studies on the effect of spiritual intelligence training on the competence of spiritual care is recommended with more samples and a larger setting.

### Prevalence to clinical practice

One of the aspects that nurses must pay special attention to regarding patient care is to improve the spiritual needs of patients. However, according to the findings of the studies on nurses’ knowledge deficiency [30–28] and the findings of this study, it is necessary to emphasize the positive effect of spiritual intelligence training on nursing education. In this regard, the inclusion of spiritual intelligence and spiritual care issues in nursing undergraduate curriculum training and in-service training of nurses are necessary. Spiritual learning training can be a valuable way to increase nurses’ attention to this aftercare.

## Acknowledgment

The authors would like to thank all the nurses participating in this research as well as the research unit of LUMS.

This article is a part of the Master Thesis of Nursing approved by the Lorestan University of Medical Sciences. Hereby, we would like to thank the university’s deputy of Education and Research, the authorities of the research environment and all the nurses who gave their time to the researcher and participated in the study.

## Conflict of Interest

The authors confirm that there are no conflicts of interest.

### Ethical Approval

The present study was approved by the Research Ethics Committee of the Lorestan University of Medical Sciences in June 2017.

### Funding

This research was funded by grants awarded by the Lorestan University of Medical Sciences.

## Footnotes

2. Cardiac Care Units

3. Intensive Care Units
